# Off‐Label Use of Topical Ruxolitinib in Dermatology: A Systematic Literature Review and Current Perspectives

**DOI:** 10.1111/exd.70095

**Published:** 2025-04-07

**Authors:** Marco Spadafora, Serena Morsia, Vito Giuseppe Di Lernia, Shaniko Kaleci, Giovanni Pellacani, Caterina Longo

**Affiliations:** ^1^ Azienda Unità Sanitaria Locale ‐ IRCCS di Reggio Emilia, Skin Cancer Center Reggio Emilia Italy; ^2^ Department of Surgery, Medicine, Dental Medicine and Morphological Sciences University of Modena and Reggio Emilia Modena Italy; ^3^ University of Modena and Reggio Emilia Modena Italy; ^4^ Dermatology Unit Arcispedale Santa Maria Nuova, Azienda USL‐IRCCS di Reggio Emilia Reggio Emilia Italy; ^5^ Department of Clinical Internal, Anesthesiological and Cardiovascular Sciences, Dermatology Clinic Sapienza University of Rome Rome Italy

**Keywords:** dermatology, JAK inhibitors, off‐label, ruxolitinib cream, topical ruxolitinib

## Abstract

JAK inhibitors are used to treat various inflammatory skin diseases. However, systemic formulations are associated with an increased risk of major adverse events. Ruxolitinib 1.5% cream is a selective topical JAK1 and JAK2 inhibitor, which has recently been approved by EMA and MHRA for treating non‐segmental vitiligo, while being FDA‐approved for both vitiligo and atopic dermatitis. Recent literature has reported the off‐label use of topical Ruxolitinib for several skin conditions, but data are mostly limited to single case reports and series and few prospective studies, with mixed results. We conducted a systematic review of the literature to investigate the potential efficacy of topical Ruxolitinib in various skin diseases in an off‐label setting. The following keywords were used for searching the MEDLINE (Pubmed) and Scopus databases from inception to September 2024: “ruxolitinib cream and dermatology” and “topical ruxolitinib and dermatology”. Reviews, articles not focusing on the main topic, books and book chapters, and articles with no English text were excluded. A total of 170 studies were screened, of which 112 fell within exclusion criteria and 58 were assessed for eligibility. Of these, 28 studies, published between 2012 and 2024, were selected. Ruxolitinib cream resulted in being used off‐label mostly for treating lichenoid and granulomatous dermatoses, as well as alopecia areata. While for the former skin conditions, topical ruxolitinib proved to be effective and safe, results on efficacy in alopecia areata were controversial. Topical ruxolitinib might be a promising therapeutic option for lichenoid and granulomatous dermatoses. Noteworthily, despite the exciting results from the oral formulation, no consistent data were described for topical ruxolitinib in alopecia areata. Our review reported encouraging results for many inflammatory skin conditions that should be investigated in further studies.

## Graphical abstract

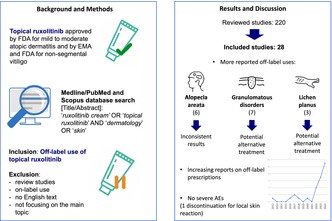

## 1 Introduction

In recent years, JAK inhibitors have been approved in many countries for the treatment of psoriasis, atopic dermatitis, alopecia areata (AA), and vitiligo, representing a promising therapeutic approach for various skin conditions [1, 2, 3]. Despite their efficacy, oral JAK inhibitors have been associated with significant adverse events, including major cardiovascular and thromboembolic events, systemic infections, and malignancies [1, 3, 4, 5]. Therefore, topical JAK inhibitors represent a promising class of medications with a lower risk of side effects and drug interactions compared to systemic formulations.

Among topical JAK inhibitors, ruxolitinib is a selective inhibitor of JAK1 and JAK2, key enzymes in skin and scalp inflammation [1]. The selective inhibition exerted by ruxolitinib leads to a decrease in downstream cytokines, including interleukins IL‐4, IL‐13, and IL‐31, as well as thymic stromal lymphopoietin (TSLP), which play pivotal roles in pruritic dermatoses [6].

To date, ruxolitinib 1.5% cream has been authorised by the Food and Drug Administration for the treatment of mild to moderate cases of atopic dermatitis and non‐segmental vitiligo in adults and adolescents aged 12 and older when other treatments have not been successful [6, 7, 8, 9]. This formulation was also recently approved by the European Medicines Agency and Medicines and Healthcare Products Regulatory Agency in the UK for the treatment of non‐segmental vitiligo [10, 11].

As previously reported for other topical JAK inhibitors [12, 13], topical ruxolitinib may demonstrate efficacy in inflammatory and immune‐mediated skin conditions other than vitiligo and atopic dermatitis.

We reviewed the literature to provide the most up‐to‐date evidence‐based data on the off‐label use of topical ruxolitinib in dermatology.

## 2 Materials and Methods

**FIGURE 1:**
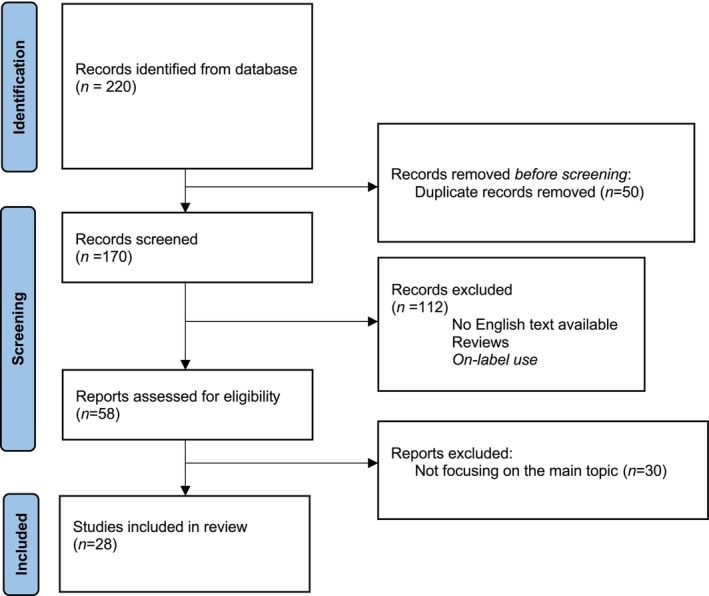
Flowchart describing the inclusion methodology applied in our review process (PRISMA flowchart).

This systematic review was based on the PRISMA guidelines. A flowchart diagram is presented in Figure 1.

### 2.1 Search Strategy

A search was conducted in the MEDLINE/Pubmed and Scopus electronic databases from inception to present (up to September 2024). The specific search strategy used the following terms for MEDLINE/PubMed databases: ‘ruxolitinib cream’ [Title/Abstract] OR ‘topical ruxolitinib’ [Title/Abstract] AND ‘dermatology’ [Title/Abstract] OR ‘skin’ [Title/Abstract]. We used the following terms for Scopus database: “ruxolitinib cream” OR “topical ruxolitinib” [Article title/Abstract/Keywords] AND “dermatology” “OR”“skin” [Article title/Abstract/Keywords]. Only English‐language publications were considered. To integrate the results from Scopus with those retrieved from MEDLINE/PubMed, we exported all records into reference management software (Zotero). During this process, duplicate records were removed both automatically and manually. We then screened the titles and abstracts according to inclusion and exclusion criteria, followed by a full‐text review of the selected articles. To address publication bias, we employed a comprehensive and systematic search strategy, which included multiple databases. This approach minimises the risk of overlooking relevant studies. Only journal articles were included in the analysis, while conference abstracts, preprint papers, books, and book chapters were omitted. Exclusion criteria involved articles that did not provide an English translation, primarily due to resource constraints of our research team on non‐English studies. Review articles were not included.

Only publications specifically addressing the off‐label use of topical ruxolitinib were considered. As extensive evidences were published about the use of ruxolitinib cream for atopic dermatitis [14, 15], articles concerning this indication, approved by the FDA but not yet in Europe or in the UK, were also excluded.

### 2.2 Study Population—Selection

The following PICO (Population, Intervention or exposure, Comparison, Outcome) elements were applied as inclusion criteria for this systematic review: (i) Population: patients affected by a skin condition, (ii) Intervention: use of topical ruxolitinib to treat a skin condition, (iii) Comparison: standard of care/therapy, (iv) Outcome: topical ruxolitinib efficacy in improving the skin condition.

### 2.3 Data Extraction

For studies meeting the inclusion criteria, two independent reviewers (S.M. and M.S.) collected data in a standardised and predefined form. Disagreements were resolved by a third reviewer (C.L.). For every paper, the following information was collected: first author, year of publication, skin condition, study design, sample size, dosage, outcome(s), adverse effect(s), concomitant therapy(ies), and previous treatment(s) (Table 1). We assessed the risk of bias in individual studies using the Risk‐of‐bias Visualisation (robvis) [16].

**TABLE 1:** List of included articles and main findings.

Author (year)	Condition	Study design	Sample size (N)	Posology	Outcome(s)	Adverse effect(s)	Concomitant therapies	Previous treatments
Olsen (2020)	Alopecia areata	Two parts, double‐blind, randomised, vehicle‐controlled phase 2 study	Part A: 12 Part B: 39	Part A: 24 weeks+24 weeks Part B: 24 weeks+24 weeks	Part A: SALT_50_ was achieved in 3 patients (25.0%) at week 12, 4 (33.3%) at week 18, and 6 (50.0%) at week 24 Part B: no significant difference in hair regrowth by SALT_50_ between patients on topical ruxolitinib and vehicle	7 patients with local reactions, 1 patient discontinued the study; No systemic reactions	Not applicable	Not applicable
Bokhari (2018)	Alopecia areata	Prospective, double‐blind placebo controlled, pilot study	16	Ruxolitinib 1% ointment BID for 12 weeks	Hair regrowth described in 5/16 in ruxolitinib, 6/16 in tofacitinib and 10/16 in the clobetasol treated area; areas treated with 0.05% clobetasol showed the most significant regrowth	No AEs	Not applicable	Not applicable
Bayart (2017)	Alopecia areata	Case series	2	Case 1: Ruxolitinib 2% in liposomal base to eyebrow regions BID Case 2: Ruxolitinib 1% in liposomal base to eyebrow regions and upper eyelids BID × 18 months Severity of Alopecia Tool	Case 1: no effects Case 2: upper lashes regrowth up to 75%; no regrowth of eyebrows	Not applicable	Not applicable	Case 1: topical and intramuscular corticosteroids, SADBE Case 2: topical and intralesional corticosteroids
Craiglow (2016)	Alopecia areata	Case report	1	Ruxolitinib 0.6% cream BID for 12 weeks	Eyebrows almost fully regrowth and a 10% increase in hair of the scalp	No AEs	Not applicable	Prednisone Intralesional Triamcinolone Sulfasalazine Topical squaric acid dibutylester Topical anthralin
Deeb (2017)	Alopecia areata	Case report	1	Ruxolitinib 0.6% cream QD for 8 weeks then BID for 12 weeks	Treatment was unsuccessful (SALT score 29 a the end of systemic therapy to 27 at the end of ruxolitinib)	No AEs	Not applicable	Clobetasol solution Minoxidil 5% foam Injections with Triamcinolone acetonide Oral prednisone 40 mg Cyclosporine 3 mg/kg divided into bid—oral methotrexate 15–25 mg per week
Tembunde (2024)	Alopecia areata	Case report	1	BID for 4 weeks and then retreatment for a 24 weeks course	After first treatment hair regrowth was noticed in alopecic patches, despite one and a new alopecic patch was discovered; after the retreatment all alopecic patches resolved	No AEs	Fluocinonide 0.05% solution QD (only during first treatment) Oral fexofenadine 30 mg BID (only during first treatment)	Clobetasol 0.05% cream
Desai (2024)	Frontal fibrosing alopecia	Case report	1	QD for 12 weeks	Clearance of facial papules, resolution of subjective symptoms and stabilisation of the frontal hairline	No AEs	Topical minoxidil 5% solution Oral minoxidil 5 mg/days Dutasteride 0.5 mg/days Excimer narrow band UV‐B laser	Doxycycline hyclate 100 mg BID Hydroxychloroquine 200 mg BID—intralesional triamcinolone injections Tacrolimus 0.3% mixed with Cetaphil cleaner Topical clobetasol 0.05% solution Pioglitazone 15 mg/d
Brumfiel (2022)	Lichen planus (classic form)	Prospective phase II study on 12 patients	12	BID for 8 weeks	Total lesion count decreased by a median of 50 lesions (median lesion count before treatment 75; *p* < 0.001); Modified Composite Assessment of Index Lesion Severity scores decreased by a mean difference of 7.6	Only one possibly related AE (abnormal taste)	Not applicable	All subjects was first treated with topical corticosteroids and other topical or systemic therapies depending on the single cases
Min (2024)	Liche planus (multivariant form)	Case report	1	BID	Clearence of oral mucosal lesions	No AEs	Baricitinib 2–4 mg QD—topical corticosteroids—prednisone tapers	Clobetasol 0.05% ointment—triamcinolone 0.1% ointment—oral prednisone—oral mycophenolate—betamethasone 0.05% ointment, —intramuscular triamcinolone injections—adalimumab
Cornman (2023)	Lichen planus (pigmentosus form)	Case report	1	BID for 27 weeks	Improvement of hyperpigmentation and pruritus (WI‐NRS score 2/10)	No AEs	Photoprotection	Topical steroids Topical calcineurin inhibitors Ketoconazole cream Topical hydroquinone Oral minocycline
Zundell (2024)	Lichen sclerosus et atrophicus	Case report	1	BID for 6 weeks	Improvement of pruritus, dysuria and erythema	Not applicable	Not applicable	High potency topical steroids Crisaborol Tacrolimus ointment
Zundell (2024)	Morphea	Case report	1	BID for 6 weeks	Improvement of pruritus, hyperpigmentation, and erythema.	Not applicable	Not applicable	Topical clobetasol Topical calcipotriene
Piontkowski (2024)	Granuloma annulare	Case report	1	BID for 12 weeks	Resolution of GA lesions	No AEs	Not applicable	Betamethasone dipropionate 0.05% cream BID Tacrolimus 0.1% ointment BID Intralesional triamcinolone injections Hydroxychloroquine 200 mg BID
Gorham (2023)	Lupus miliaris disseminatus faciei	Case report	1	BID for 12 weeks	75% of facial papules clearing up after 1 month and further progression up to almost complete clearance at 3 months	No AEs	Not applicable	Ivermectin 1% cream QD Doxycycline 100 mg BID
Hwang (2024)	Necrobiosis lipoidica	Prospective single‐arm, open‐label, phase 2 trial	12	BID for 12 weeks	Reduction of mean lesion score from 4.4 to 1.7 (*p* < 0.003) and Skindex‐16 scores improved from 33.8 to 11.8 (*p* < 0.003); no significant effects on total body lesion count or pruritus	3 probably/possibiy mild AEs related to ruxolitinib	Not applicable	Topical corticosteroids (91.7% of subjects)
Nugent (2022)	Necrobiosis lipoidica	Case report	1	BID for 12 weeks	Improvement in colour and size of lower extremity plaques	Not applicable	Pentoxifylline 400 mg BID	High‐potency topical steroids Pimecrolimus cream Hydroxychloroquine Methotrexate—tofacitinib 2.0% cream BID
Smith (2023)	Cutaneous sarcoidosis	Case report	1	BID for 6 weeks	Complete resolution of plaque on the forehead	Not applicable	Infliximab 10 mg/kg every 4 week‐methotrexate 25 mg subcutaneous weekly	Low‐potency topical steroids Intralesional triamcinolone acetonide at 5 mg/mL
Wong (2023)	Granuloma faciale	Case report	1	BID for 12 weeks	Improvement with flattening of the plaque	No AEs	Not applicable	Intralesional triamcinolone 5 mg/cc every 6 weeks Tacrolimus ointment 0.1% BID
Zhang (2023)	Granulomatous scleromyxedema	Case report	1		Improvement of pruritus decrease of redness and induration	Not applicable	Oral dapsone 100 mg IVIG 2 g/kg	Topical triamcinolone Oral prednisone Narrow‐band UV‐B Administered in divided doses every 28 days
Punwani (2012)	Plaque psoriasis	Randomised controlled trial	27	BID for 4 weeks	Compared to vehicle, Ruxolitinib 1.5% cream resulted in improvements in lesion thickness, erythema, and scaling, along with a reduction in lesional area.	Mild to moderate local AEs, no treatment discontinuation	Not applicable	Not applicable
Zundell (2024)	Notalgia paresthetica	Case report	1	BID for 6 weeks	Pruritus improvement	Not applicable	Camphor Menthol lotion Amlactin lotion	Topical triamcinolone Lidocaine patches
Shin (2024)	Tattoo pruritus	Case series	2	Case 1: ruxolitinib 1.5% cream for 5 weeks Case 2: ruxolitinib 1.5% cream for 14 weeks	Case 1: resolution of pruritus and erythematous plaque Case 2: resolution of pruritus and sliglhty reduction of nodules	Not applicable	Case 1: ‐ Case 2: intralesional triamcinolone	Case 1: ‐ Case 2: Clobetasol 0.05% ointment wraps
Park (2022)	Discoid lupus erythematosus	Case report	1	QD for 8 weeks	Improvement of scalp plaque and subtle hair regrowth	Not applicable	Belimumab	Hydroxychloroquine 400 mg daily Halobetasol 0.05% ointment
Pope (2022)	Seborrheic dermatitis	Case report	1	BID for 8 weeks	Complete resolution of seborrheic dermatitis and partial improvement of rosacea	Not applicable	Not applicable	Ketoconazole cream Desonide cream Hydrocortisone cream Metronidazole 1% gel Doxycycline 100 mg
Teklu (2023)	Seborrheic dermatitis	Case report	1	Every other day for 4 weeks	Almost complete resolution	Not applicable	Not Applicable	Ketoconazole cream BID for 2 month Zinc pyrithione shampoo 3 times a week Betamethasone cream BOD for 1 week per month Ketoconazole cream QD Oral fluconazole 200 mg weekly Azelaic acid 15% gel BID Pimecrolimus cream BID Ivermectin 21 mg weekly for 4 weeks Topical hydrocortisone 2.5% Triamcinolone 0.1% Ciclopirox shampoo
Tran (2024)	Perioral dermatitis	Case report	1	BID for 34 weeks	Resolution of skin papules and normalisation of skin pigmentation	No AEs	Not applicable	Triamcinolone 0.1% ointment Pimecrolimus 1% cream Amoxicillin/clavulanate 875/125 mg BID (S. aures sovrainfection) Mupirocin 2% ointment BID (S. aures sovrainfection)
Zundell (2024)	Perioral dermatitis	Case report	1	BID for 4 weeks	Resolution of perioral dermatitis	Not applicable	Not applicable	Topical tacrolimus Ketoconazole Pimecrolimus Desonide Crisaborole Nystatin Hydrocortisone
Zarowin (2024)	Facial blaschkitis	Case report	1	BID for 8 weeks	Almost complete resolution of the erythematous plaque	Not applicable	Not applicable	Pimecrolimus 1% cream Tacrolimus 0.03% ointment Fluocinolone 0.025% ointment Fluocinonide 0.05% cream Calcipotriene 0.005% cream—CO_2_ laser
Powers (2024)	Immune checkpoint inhibitor‐induced eczematous reaction	Case report	1	BID for nearly 4 weeks, then applied on the day of infusions and once daily for 1 week thereafter	Near‐total facial and upper limbs rash resolution and pruritus clearance	No AEs	Pembrolizumab every 3 weeks—nab‐paclitaxel	Betamethasone dipropionate 0.05% ointment Triamcinolone 0.1% lotion BID Hydrocortisone 2.5% cream BID
Khang (2023)	Hailey‐Hailey disease	Case report	1	BID for 4 weeks	Complete resolution of residual inguinal lesions	No AEs	Dupilumab 300 mg/2‐mL subcutaneous every 14 days	Topical tacrolimus Oral antibiotics Topical erythromycin Intralesional steroids Topical steroids Apremilast
Shea (2024)	Topical steroid withdrawl	Case report	1	QD for 12 weeks	Substantial improvement of erythema and plaque induration	Not applicable	Not applicable	Doxycycline 20 mg QD Tacrolimus 0.1% ointment BID Pimecrolimus cream

## 3 Results

The bibliographic research performed with the specified criteria identified 220 publications. 170 papers were not included after duplicate removal. There were no other systematic reviews or meta‐analyses available on the topic using the mentioned keywords. Excluding papers not meeting inclusion criteria (formatting, study design, on‐label use, not focusing on the main topic), 28 papers were considered for this review (Figure 1).

The results of the risk of bias assessment revealed that most studies had a high risk of bias, primarily due to their design (case reports or case series), lack of randomization, and limited blinding. The majority of studies did not provide high‐quality information regarding random sequence generation, allocation concealment, or blinding of outcome assessors. Only a few studies [17, 18] demonstrated more rigorous methodologies (RCTs) and were assessed as having a low risk of bias across all domains.

**FIGURE 2:**
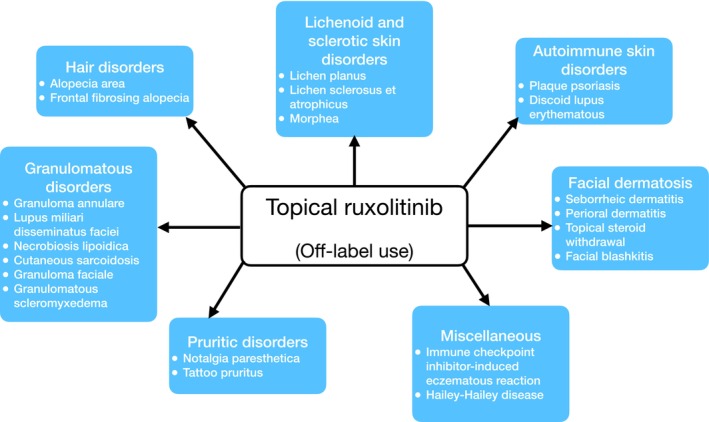
Summary of skin conditions included in the analysis, categorised by the pathogenic mechanism.

**FIGURE 3:**
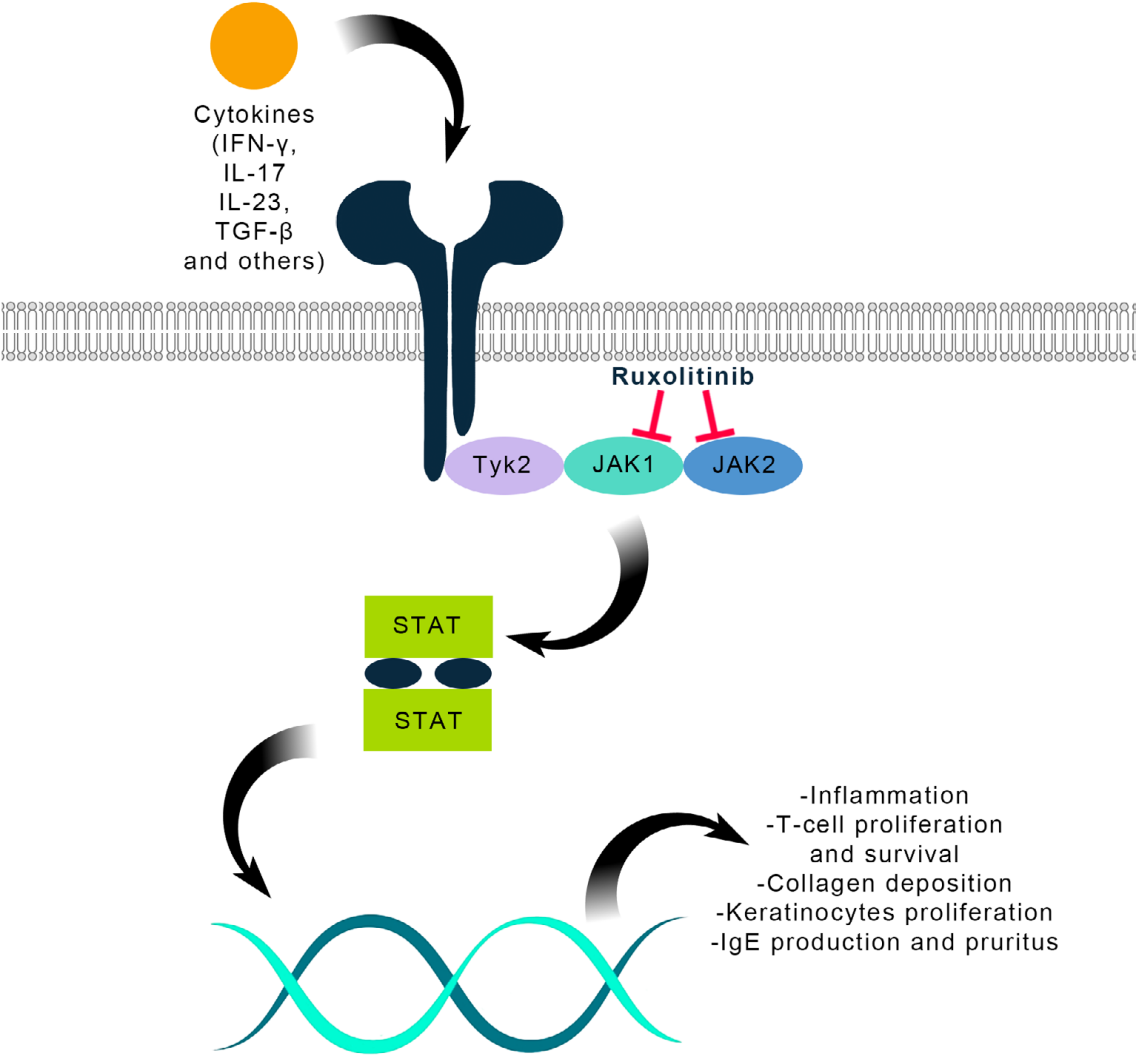
Ruxolitinib mechanism of action on the JAK–STAT pathway.

Table 1 provides a more comprehensive overview of the included articles, while Table 2 summarises the reviewed skin conditions with efficacy, safety, and limitations data. In Figure 2, a summary of skin conditions categorised by their pathogenic types is presented. Figure 3 provides an overview of the mechanism by which Ruxolitinib selectively inhibits the JAK–STAT signalling pathway. We present the key outcomes of our research organised by skin condition.

**TABLE 2:** Efficacy, safety, and limitations in reviewed skin conditions of topical ruxolitinib.

Condition	Nr. studies	Efficacy	Safety	Limitations
Alopecia areata	6	Inconsistent results across different studies evaluating scalp lesions: from almost fully regrowth of the hair in alopecic patches to no effect; inconsistent results also on eyebrows and lashes alopecic areas.	No systemic reactions reported; mainly local reaction	Consider as a confounders the spontaneous resolution of alopecica areata patches
Frontal fibrosing alopecia	1	Clearance of facial papules with stabilisation of frontal hairline; resolution of symptoms	No AEs	Single case report; concomitant therapies (oral and topical minoxidil, oral dutasteride, excimer laser)
Lichen planus	3	Total lesion count reduction (classic variant), resolution of pruritus and hyperpigmentation (pigmentosus variant) and on oral mucosal lesions (multivariant form)	Reported one case of abnormal taste	Concomitant use of oral baricitinib in the oral variant
Lichen sclerosus et atrophicus	1	Improvement of pruritus, dysuria and erythema on the genital area	N/A	Single case report
Morphea	1	Improvement of pruritus, hyperpigmentation, and erythema.	N/A	Single case report
Granuloma annulare	1	Clearence of the lesions	No AEs	Single case report
Lupus miliaris disseminatus faciei	1	Almost complete clearance of manifestation	No AEs	Single case report
Necrobiosis lipoidica	2	Reported effects were a reduction in mean lesion score in specific body site and improvement in colour and size in lower limbs	Mild local AEs	Two case reports; no significant effects on total body lesion count or pruritus; in a single case confounder of concomitant use of systemic pentoxifylline
Cutaneous sarcoidosis	1	Clearence of sarcoidoisis cutaneous manifestation	N/A	Single case report
Granuloma faciale	1	Flattening of the plaque	No AEs	Single case report; concomitant use of systemic infliximab and methotrexate
Granulomatous scleromyxedema	1	Improvement of pruritus, redness, and induration of the manifestation	N/A	Single case report; concomitant use of oral dapsone and IVIG
Plaque psoriasis	1	Improvements in lesion thickness, erythema, and scaling, and reduction in lesional area.	Mild to moderate local AEs, no treatment discontinuation	
Notalgia paresthetica	1	Imrovement of pruritus	N/A	Single case report
Tattoo pruritus	1	Pruritus resolution with complete to partial clearence of the lesions	N/A	Two case reports; in a single case concomitant use of intralesional triamcinolone
Discoid lupus erythematosus	1	Improvement of scalp plaque and subtle hair regrowth	N/A	Single case report; concomitant use of systemic belimumab
Seborrheic dermatitis	2	Complete/almost complete clearence of seborrheic dermatitis	N/A	Two case reports
Perioral dermatitis	2	Clearence of facial papules and resolution of hyperpigmentation	No AEs	Two case reports
Facial blaschkitis	1	Almost complete resolution of the erythematous plaque	N/A	Single case report
Immune checkpoint inhibitor‐induced eczematous reaction	1	Rash and pruritus resolution	No AEs	Single case report
Hailey‐Hailey disease	1	Complete resolution of lesions not responding to systemic therapy	No AEs	Single case report
Topical steroid withdrawl	1	Improvement of erythema and induration	N/A	Single case report

### 3.1 Alopecia Areata

Six studies investigated the efficacy of topical ruxolitinib in the management of AA.

A 2‐parts, double‐blind, randomised, vehicle‐controlled phase 2 study was conducted on patients with AA with at least 25% hair loss by Severity of Alopecia Tool (SALT) score [17]. Part A of the study examined the effects of 1.5% ruxolitinib cream for 24 weeks, with an additional 24‐week period. Part B was a double‐blind study of 24 weeks on 1.5% ruxolitinib cream versus vehicle and a further 24‐week period where the vehicle group was switched to ruxolitinib cream. Efficacy was assessed by a 50% improvement in SALT score. Part A showed efficacy of 1.5% ruxolitinib cream with a SALT_50_ (≥ 50% improvement from baseline in SALT) achieved by 50% of patients at week 24. Conversely, in Part B, there was no significant difference in hair regrowth comparing ruxolitinib group data to the vehicle group.

A prospective, double‐blind placebo‐controlled study tested the efficacy of ruxolitinib 1% ointment, tofacitinib 2% ointment, clobetasol dipropionate 0.05% ointment, and vehicle (ointment) on different areas of 12 subjects affected by alopecia uiniversalis [12]. Hair regrowth was described in 5/16, 6/16, and 10/16 subjects, respectively. Despite the efficacy of all the active treatments, overall hair regrowth was better in the clobetasol‐treated areas.

A case series reported data from 6 cases of paediatric AA treated with topical JAK inhibitors [13]. In 2 reports, patients were treated with topical ruxolitinib. The first patient was a 4‐year‐old boy who applied ruxolitinib 2% in liposomal base on his eyebrows. No regrowth was assessed. The second patient was a 17‐year‐old girl treated with ruxolitinib 1% in liposomal base for AA of her eyebrows and upper eyelids. It was described that ∼75% of upper eyelashes regrew, but no regrowth of eyebrows occurred.

A further case report described a young woman in her late teens affected by alopecia universalis, recalcitrant to treatments [19]. After topical use of ruxolitinib 0.6% cream, she experienced an almost full regrowth of her eyebrows and 10% regrowth of the hair of the scalp.

A second case report showed the failure of topical ruxolitinib on AA in a 66‐year‐old woman with a relapsing–remitting diease [20]. No SALT score improvement was assessed after switching her therapy to ruxolitinib 0.6% cream.

A recent case report described topical ruxolitinib use on a 5‐year‐old girl treated with ruxolitinib 1.5% cream [21]. After the first treatment compounded with fluocinonide solution and oral fexofenadine, hair regrowth was reported in almost all alopecic patches. After retreatment with only topical ruxolitinib for a longer period, a full hair regrowth was described. Although strong pathogenetic evidence supported the use of ruxolitinib in AA due to its ability to inhibit signalling from both the γc cytokines (JAK1/JAK3) and interferon‐gamma (IFN‐γ) signalling (JAK1/JAK2) in managing AA [22, 23, 24, 25], no conclusive data support the use of topical drugs [13, 17, 19, 20, 21, 26, 27, 28, 29].

### 3.2 Frontal Fibrosing Alopecia

A case report of a 55‐year‐old male with regression of the frontal hairline, scalp and eyebrow pruritus, tenderness, and facial papules showed that using ruxolitinib 1.5% cream determined a clearance of papules, a stabilisation of the frontal hairline, and a resolution of symptoms like itching and burning [30]. Although the specific mechanisms of FFA are yet to be determined [31], the potential therapeutic role of ruxolitinib is suggested by the activation of IFN‐γ signalling [32] and expression of JAK proteins in inflammatory cells in FFA [33]. Adjunctively, the shared inflammatory pathogenesis of FFA with vitiligo supports the potential efficacy of topical ruxolitinib [34, 35].

### 3.3 Lichen Planus

Three studies outlined the potential application of topical ruxolitinib in lichen planus (LP).

A prospective phase II study enrolled 12 patients with classic and hypertrophic LP variants [36]. The study showed a significant decrease in total lesion count (from a median lesion count of 75 to a median lesion count of 50, *p* < 0.001) and improvement in modified Composite Assessment of Index Lesion Severity scores.

Further, a case report of a 63‐year‐old male affected by a refractory form of LP pigmentosus on his cheeks showed first a stabilisation and then a complete resolution after 9 months of topical ruxolitinib [37].

A second case report [38] described a 61‐year‐old woman with both cutaneous and mucosal lichen planus recalcitrant to first‐ and second‐line systemic and topical therapies. The patient was first started on oral baricitinib with improvement of skin manifestation but with no efficacy on mucosal lesions. Interestingly, erosive oral LP improved only after the adjunctive therapy of ruxolitinib cream.

The JAK2/STAT1 pathway takes part in the pathogenesis of LP as previously described [39, 40], supporting the use of topical ruxolitinib in LP management.

### 3.4 Lichen Sclerosus et Atrophicus

A single case from a case series reported the efficacy of topical ruxolitinib in a paediatric lichen sclerosus et atrophicus (LSA) [41]. A 9‐year‐old female manifested atrophy and hypopigmentation of labia minora and major and erythema from vulva to anus. After starting on ruxolitinib 1.5% cream, she experienced an improvement in the erythematous patch, pruritus, and dysuria. Activation of the Th1 response and elevation of pro‐inflammatory cytokines were connected to LSA pathogenesis [42]. The hypothesized role of topical ruxolitinib in LSA is to reduce the inflammatory response via JAK/STAT pathway inhibition, thereby preventing the production of collagen and the onset of fibrosis [41].

### 3.5 Morphea

Topical ruxolitinib was found to be effective in treating morphea in a single patient within a case series [41]. A 13‐year‐old girl suffering from insulin‐dependent diabetes was diagnosed with morphea manifesting as pruritic depressed hyperpigmented plaques on her bilateral shins. The affected areas were treated with ruxolitinib 1.5% cream with improvement in her pruritus, hyperpigmentation, and erythema. Morphea was previously demonstrated to be an inflammatory‐driven fibrosis [41]. As described for oral JAK inhibitors [42, 43], ruxolitinib may inhibit pro‐inflammatory cytokines production. JAK2 and STAT3 have been recognised as key players in this disease pathway, leading to evidence that blocking JAK2 can reduce transcription of genes that promote fibrosis [44, 45].

### 3.6 Granuloma Annulare

A single case report described a 51‐year‐old female with granuloma annulare (GA) affecting the face and lower extremities, not responsive to topical standard therapeutics nor to systemic hydroxychloroquine [46]. After 3 months of treatment with ruxolitinib 1.5% cream, she obtained a complete clearance of GA. It was previously demonstrated the involvement of JAK pathways in GA pathogenesis [47, 48] but to date, only systemic JAK inhibitors have been reported in the management of GA.

### 3.7 Lupus Miliaris Disseminatus Faciei

In a single report [49], the case of a 46‐year‐old woman with a worsening eruption on her face was described, not responsive to systemic or topical first‐line treatments. As the patient refused to start on new systemic medications, ruxolitinib 1.5% cream was proposed. After 12 weeks, it was documented an almost complete clearance of facial papules. As previously hypothesised, the blockage of inflammation driven by JAK/STAT signalling appeared to be a key mechanism to exert the efficacy of topical ruxolitinib in granulomatous diseases [50].

### 3.8 Necrobiosis Lipoidica

Two different studies described the effects of topical ruxolitinib on necrobiosis lipoidica (NL).

A prospective single‐arm, open‐label trial enrolled 12 patients with a history of refractory NL. After applying ruxolitinib 1.5% cream, a significant improvement was reported by mean NL lesion score and Skindex‐16 score, while no significant effects on total body lesion count or pruritus numerical rating scale were registered [51].

A case report [52] described a 19‐year‐old woman with a history of NL on the shins recalcitrant to systemic and topical therapies. A partial improvement was documented after using systemic pentoxifylline, hydroxychloroquine, and tofacitinib 2.0% cream, while dramatic improvement was reported after switching to ruxolitinib 1.5% cream. The authors hypothesised that the greater efficacy of topical ruxolitinib compared to topical tofacitinib might be due to different molecular targets (JAK1‐2 and JAK1‐3 respectively). Adjunctively, the tofacitinib compounded formulation might have influenced the therapeutic result [52].

The efficacy of JAK/STAT inhibitors was also confirmed in different studies on NL [53, 54, 55, 56]. As with other granulomatous disorders, NL is also caused by an inflammatory response via JAK/STAT signalling [50].

### 3.9 Sarcoidosis

In a case report, a 66‐year‐old woman with a history of multisystem sarcoidosis presented with an indurated pink plaque on the left side of her forehead recalcitrant to systemic and topical treatments [57]. After adjunctive treatment with ruxolitinib 1.5% cream, a complete resolution of her plaque was described. Previous studies showed the influence of JAK/STAT signalling on the pathogenesis of cutaneous sarcoidosis [47, 58, 59].

### 3.10 Granuloma Faciale

Topical ruxolitinib efficacy was described on a 75‐year‐old female presenting with a plaque of her forehead biopsy‐diagnosed as granuloma faciale [60]. After ruxolitinib cream application, the patient experienced a significant improvement. As high levels of interleukin 5 and interferon‐gamma expression were described in GF [61, 62], JAK‐inhibitors efficacy is probably caused by a reduction of these pro‐inflammatory citokines [60].

### 3.11 Granulomatous Scleromyxedema

A man in his 60s presenting with papules, erythema, and induration of the skin on almost the whole body surface was diagnosed with granulomatous scleromyxedema and underlying monoclonal gammopathy recalcitrant to many treatments. After switching to oral dapsone and IvIG plus ruxolitinib 1.5% cream on the most bothersome area of his body, the patient reported improvement of pruritus, redness, and induration. It was previously demonstrated in vitro fibroblast proliferation in the serum derived from a scleromyxedema [63]. As a key mechanism, the authors hypothesized a possible anti‐inflammatory/anti‐fibrotic activity of topical ruxolitinib [64].

### 3.12 Plaque Psoriasis

A randomised proof of control trial [18] tested the efficacy of ruxolitinib 1.5% cream compared to vehicle and active comparators (calcipotriene 0.005% cream or betamethasone dipropionate 0.05% cream) on 27 subjects affected by plaque psoriasis. After applying ruxolitinib 1.0% cream daily for 28 days, the total composite lesion score of erythema, thickness, and scaling decreased by 53% (*p* = 0.033), surpassing the 32% reduction observed in lesions treated with the vehicle. Lesions treated with 1.5% cream twice a day showed a 54% improvement compared to 32% with the vehicle (*p* = 0. 056).

A variety of cytokines that play a role in psoriasis development, including IL‐23 and IFN‐γ, rely on JAKs signalling, suggesting that JAK inhibition could be an effective role in the treatment of the disease [65]. In fact, topical ruxolitinib, as a JAK1‐JAK2 selective inhibitor, decreased the level of IL‐12 and IL‐23 [66]. Furthermore, the JAK–STAT inhibition might prevent the release of cytokines related to keratinocyte proliferation [18].

### 3.13 Notalgia Paresthetica

A case report from a series described topical use of ruxolitinib in a 72‐year‐old female HIV‐positive with pruritic notalgia paresthetica resistant to previous treatments. The affected area was treated with ruxolitinib 1.5% cream, leading to itch redcution. The efficacy of JAK inhibitors on itch has already been described for atopic dermatitis [67].

### 3.14 Tattoo Pruritus

A case series reported the efficacy of ruxolitinib 1.5% cream in treating tattoo pruritus [68]. A 30‐year‐old woman presented with pruritic plaques on the tattoo of her calf. She experienced pruritus resolution and erythematous plaque improvement after introducing topical ruxolitinib. The second case described a 47‐year‐old woman with pruritus and inflammatory nodules on two tattoos on her back and left forearm. Ruxolitinib 1.5% cream was started concomitantly with intralesional triamcinolone injections, with resolution of pruritus and improvement of nodules. As itch response is part of the signalling that JAK1 and JAK2 regulate, topical ruxolitinib manifested its efficacy through the inhibition of this pathway [68].

### 3.15 Discoid Lupus Erythematosus

Ruxolitinib 1.5% cream efficacy on discoid lupus erythematosus (DLE) was reported in a single case studyof a 28‐year‐old‐woman that experienced a right‐sided scalp patchy alopecia [69]. After starting on ruxolitinib 1.5% cream on her right scalp, the patient manifested hair regrowth in the alopecic area. It was demonstrated that skin lesions of individuals with subacute CLE and chronic DLE have higher levels of phosphorylated JAK1 [70]. Similarly, a case report detailed 2% tofacitinib ointment, a topical JAK1/3 inhibitor, being an effective treatment in periorbital DLE [71].

### 3.16 Seborrheic Dermatitis

Two case reports showed the efficacy of topical ruxolitinib on seborrheic dermatitis (SB). The first one reported the case of a 74‐year‐old man with a history of recalcitrant seborrheic dermatitis and concomitant rosacea [72]. The application of 1.5% ruxolitinib cream led to a resolution of the skin manifestations.

A second case report demonstrated the efficacy of ruxolitinib cream in a 27‐year‐old‐man with a form of SB recalcitrant to different topical and systemic therapies [73]. After switching to ruxolitinib 1.5% cream, the patient experienced an almost complete clearance.

The pathogenesis of SB is driven by IL‐4 and IL‐17, which could account for the therapeutic impact of ruxolitinib cream on this condition [74].

### 3.17 Perioral Dermatitis

Two case reports described the efficacy of ruxolitinib 1.5% cream in the management of granulomatous perioral dermatitis (PD).

A 18‐year‐old woman presented with a facial rash around her mouth and lower eyelids after topical steroid use to manage her atopic dermatitis [75]. After diagnosing a *S. aureus* sovrainfection of the area, the patient was started on amoxicillin‐clavulanate and topical mupirocin, with resolution of the sovrainfection but residual PD. Thus, the patient started ruxolitinib 1.5% cream, with improvement after 1 week and complete resolution after 34 weeks.

A case report from a case series reported the case of a 26‐year‐old woman affected by PD [41]. The identification of allergens during patch testing resulted in the necessity to avoid certain products, although the rash persisted. Following ruxolitinib 1.5% cream prescription, perioral dermatitis resolved.

The hypothesized upregulation of JAK‐signal transducer and activator of transcription pathway after prolonged application of topical steroids [75], lay a rationale for the use of topical ruxolitinib.

### 3.18 Facial Blaschkitis

A single case report described the use of ruxolitinib 1.5% cream on a rare case of facial blaschkitis [76]. A 15‐year‐old female with Down syndrome presented with an erythematous blaschkolinear plaque on the chin, recalcitrant to many topical treatments and laser therapy. After switching to topical ruxolitinib, the patient's skin manifestation was almost resolved. A CD8+ T cell infiltrate was previously described in blaschkitis specimens [77], thus the rationale behind the use of JAK inhibitors with a known activity on T‐cell mediated skin disorders.

### 3.19 Immune Checkpoint Inhibitor‐Induced Eczematous Reaction

A single case report described using topical ruxolitinib on immune checkpoint inhibitor‐induced eczematous reaction [78]. A 21‐year‐old woman tested BRCA1‐positive was diagnosed with left triple‐negative breast cancer and right ductal carcinoma in situ. She initiated pembrolizumab while undergoing cyclophosphamide and doxorubicin, followed by weekly paclitaxel and carboplatin. From the sixth cycle of pembrolizumab, the patient manifested a pruritic, erythematous rash recalcitrant to topical corticosteroids on her upper extremities, face, and neck. She was switched to ruxolitinib 1.5% cream with a resolution of her pruritus and skin rash clearance. The author outlined a potential mechanism of ruxolitinib cream via inhibition of the Th1 cascade, limiting the activation of the JAK/STAT pathway. In fact, an increased activation of Th1‐related genes was described in skin samples of cutaneous immune‐related adverse reactions [79].

### 3.20 Hailey‐Hailey Disease

A single‐case report described a 53‐year‐old woman with a history of axillae and inguinal folds Hailey‐Hailey disease (HHD) recalcitrant to several topical and systemic treatments. After starting on dupilumab, an improvement was reported in axillae manifestations, but inguinal folds disease had no response. The association of ruxolitinib 1.5% cream caused an improvement in her residual disease [80].

Topical ruxolitinib probably inhibited cytokine‐driven pathways usually activated from the loss of cellular adhesion. As a consequence, we assist in a reduced lymphocytic infiltration; a possible enhancement of cellular adhesion by dupilumab itself has also been hypothesized [80, 81, 82].

### 3.21 Topical Steroid Withdrawal

A case report described the case of a 69‐year‐old woman suffering from chronic dermatitis affecting the perioral area, caused by chronic use of topical hydrocortisone to self‐manage a contact dermatitis [83]. The manifestation persisted after discontinuing allergens that resulted positive in patch testing. As calcineurin inhibitors and oral doxycycline showed no efficacy, the patient was switched to daily ruxolitinib 1.5% cream with marked improvement. This case suggested that by the use of JAK inhibitors, the risk of a rebound cytokine release due to topical steroid withdrawal could be prevented [83].

## 4 Discussion

According to reviewed publications, off‐label prescriptions of topical ruxolitinib are increasing.

Data were mostly retrieved from single case reports and series, with few prospective and controlled studies. A variety of skin disorders and different efficacy outcomes were described. As previously reported [1], topical ruxolitinib may exert its efficacy in several inflammatory skin diseases as a consequence of high drug concentrations reached by topical JAK inhibitors in the epidermal and dermal layers.

Compared to topical corticosteroids, the most prescribed topical anti‐inflammatory drugs, which exert their efficacy through broad anti‐inflammatory action by suppressing multiple inflammatory pathways, ruxolitinib offers a more targeted approach. Prolonged use of medium‐ to high‐potency corticosteroids is associated with significant side effects, such as skin atrophy [84]. In contrast, ruxolitinib demonstrated only mild and transient side effects, such as application‐site reactions, making it an interesting alternative treatment. Although ruxolitinib demonstrated overall efficacy in treating difficult‐to‐manage skin conditions, comparative data with other topical medications or with other JAK/STAT inhibitors are limited.

The most reported experimental application of topical ruxolitinib was AA. Despite promising results from the oral formulation of ruxolitinib in AA [23], no consistent data were described for its topical formulation. A double‐blind vehicle‐controlled trial demonstrated no significant difference between ruxolitinib and placebo [17], while a comparative double‐blind controlled trial described the lower efficacy of topical ruxolitinib compared to topical tofacitinib and topical clobetasol [12]. On the other hand, single‐case reports described discrepant results, from no effect on the alopecic area to almost complete hair regrowth. Thus, data on efficacy in improving disease manifestations were only reported in low‐quality evidence studies [13, 19, 20, 21]. The inefficacy of topical ruxolitinib was attributed by some authors to a decreased drug penetration into the hair follicle [17]. Adjunctively, the unpredictable course of AA may create a bias in the assessment of the real efficacy of interventions, as spontaneous improvement is not so infrequent [85].

The second most reported use of topical ruxolitinib was LP. A prospective phase II study, with a moderate strength of evidence, and two case reports demonstrated promising results of topical ruxolitinib with significant reductions in lesion counts and symptom improvement in both cutaneous and mucosal LP variants [36, 37, 38]. Notably, systemic JAK inhibitors have been recently identified as a potential treatment option for recalcitrant LP, given their capacity to influence the progression of the disease [86, 87, 88].

Further, many studies described the efficacy of topical ruxolitinib in granulomatous disorders [46, 51, 52, 57, 60]. According to molecular studies, the primary pathogenic event in granulomatous disorders such as GA and sarcoidosis involves the dysregulation of JAK–STAT cytokines. Data indicate that topical ruxolitinib could represent a viable treatment for granulomatous skin conditions [47].

The use of topical ruxolitinib in psoriasis resulted in a notable improvement in disease manifestations that exceeded the effects of a placebo and was comparable to those of an active comparator, as demonstrated by high‐quality evidence from a randomised controlled trial [18].

Single‐center experiences of topical ruxolitinib as an experimental application were also reported for common skin diseases such as SLE, seborrheic dermatitis, perioral dermatitis, morphea, and lichen sclerosus, with promising results. Previous studies confirmed the potential influence of JAK inhibitors in many of these skin conditions [89, 90].

For these common skin diseases, the efficacy of topical ruxolitinib needs to be established through further prospective studies involving a larger cohort of subjects, as these conditions are frequently observed.

Our research also outlined off‐label prescription of topical ruxolitinib for uncommon skin conditions with unmet therapeutic needs such as notalgia paresthetica, topical steroid withdrawal, scleromyxedema, Hailey‐Hailey disease, or immune checkpoint inhibitor‐induced eczematous reaction. Although infrequent conditions present challenges for conducting large cohort studies, single case data suggested a potential use of ruxolitinib in managing the most bothersome, recalcitrant forms.

No severe adverse effects were observed in the reviewed studies. Further, adverse events were rare and mild, mainly localised skin reactions. Notably, only one subject discontinued an AA study because of a localised skin reaction [17].

Our study is limited by the inclusion of various study designs, impacting the level of evidence obtained. Topical ruxolitinib formulations were not homogeneous among different studies, mainly before ruxolitinib 1.5% cream was placed on the market for AD and vitiligo. Most of the studies prescribed ruxolitinib 1.5% cream, although different vehicles (ointments) and concentrations (from 0.6% to 2%) were described. Additionally, we outlined a variation in dosing across different studies. Frequently, prescriptions were made on a twice‐a‐day basis, although some studies described a once‐a‐day application. Therapy duration also varies in different reports, lasting from 4 weeks to nearly 2 years. Variations in dosing regimens may result in inconsistent efficacy outcomes across different studies. This heterogeneity complicates efforts to draw definitive conclusions about the optimal dosing strategy.

Possibly, the exclusion in our research strategy of conference abstracts, pre‐print studies, and non‐English papers may have contributed to a selection bias.

A further limitation of our study is the lack of uniformity in the measure of outcomes. Different scores were applied to account for the improvement of skin conditions. Future studies should adopt validated tools to assess the efficacy of topical ruxolitinib. Finally, a possible confounder in some reports was the concomitant use of both systemic therapy and other topical therapy along with topical ruxolitinib. Further studies should clarify the possible interaction of different medications.

We underline the need for large‐scale randomised RCTs to compare different ruxolitinib dosages, to optimise treatment efficacy and to assess the long‐term safety profile ensuring risk–benefit balance.

## 5 Conclusions and Perspectives

In conclusion, ruxolitinib appears to be a promising topical medication for various dermatologic conditions. To date, there is insufficient data to assess the long‐term efficacy and safety of off‐label use. Future clinical trials should evaluate ruxolitinib cream as an alternative treatment for skin conditions that are unresponsive to first‐line therapies.

## Author Contributions

Conceptualization: Caterina Longo; data curation: Marco Spadafora and Serena Morsia; formal analysis: Marco Spadafora, Shaniko Kaleci, and Serena Morsia; supervision: Caterina Longo; investigation: Marco Spadafora and Serena Morsia; writing – original draft: Marco Spadafora, Serena Morsia, and Caterina Longo; writing – review and editing: Caterina Longo, Vito Giuseppe Di Lernia, and Giovanni Pellacani.

## Ethics Statement

EC approval was not required since human subjects were not directly involved in the present study.

## Conflicts of Interest

The authors declare no conflicts of interest.

## Data Availability

The data that support the findings of this study are available from the corresponding author upon reasonable request.

## References

[1] H. A. Miot , P. R. Criado , C. C. S. de Castro , M. Ianhez , C. Talhari , and P. M. Ramos , “JAK‐STAT Pathway Inhibitors in Dermatology,” Anais Brasileiros de Dermatologia 98 (2023): 656–677. 37230920 10.1016/j.abd.2023.03.001 PMC10404561

[2] A. Muddebihal , A. Khurana , and K. Sardana , “JAK Inhibitors in Dermatology: The Road Travelled and Path Ahead, a Narrative Review,” Expert Review of Clinical Pharmacology 16 (2023): 279–295. 36946306 10.1080/17512433.2023.2193682

[3] B. Klein , R. Treudler , and J. C. Simon , “JAK‐Inhibitors in Dermatology ‐ Small Molecules, Big Impact? Overview of the Mechanism of Action, Previous Study Results and Potential Adverse Effects,” Journal der Deutschen Dermatologischen Gesellschaft 20 (2022): 19–24. 10.1111/ddg.14668 34962052

[4] R. Wlassits , M. Müller , K. H. Fenzl , T. Lamprecht , and L. Erlacher , “JAK‐Inhibitors ‐ A Story of Success and Adverse Events,” Open Access Rheumatology: Research and Reviews 16 (2024): 43–53. 38435420 10.2147/OARRR.S436637 PMC10906274

[5] C. Samuel , H. Cornman , A. Kambala , and S. G. Kwatra , “A Review on the Safety of Using JAK Inhibitors in Dermatology: Clinical and Laboratory Monitoring,” Dermatologic Therapy 13 (2023): 729–749. 10.1007/s13555-023-00892-5 PMC9930707 36790724

[6] A. Sheikh , W. Rafique , R. Owais , F. Malik , and E. Ali , “FDA Approves Ruxolitinib (Opzelura) for Vitiligo Therapy: A Breakthrough in the Field of Dermatology,” Annals of Medicine and Surgery 81 (2022): 104499, 10.1016/j.amsu.2022.104499. 36147080 PMC9486756

[7] K. Papp , J. C. Szepietowski , L. Kircik , et al., “Efficacy and Safety of Ruxolitinib Cream for the Treatment of Atopic Dermatitis: Results From 2 Phase 3, Randomized, Double‐Blind Studies,” Journal of the American Academy of Dermatology 85 (2021): 863–872. 33957195 10.1016/j.jaad.2021.04.085

[8] A. Blauvelt , L. Kircik , K. A. Papp , et al., “Rapid Pruritus Reduction With Ruxolitinib Cream Treatment in Patients With Atopic Dermatitis,” Journal of the European Academy of Dermatology and Venereology 37 (2023): 137–146. 36066323 10.1111/jdv.18571 PMC10087253

[9] Incyte Corporation , “OPZELURA (ruxolitinib) Cream, for Topical Use: US Prescribing Information,” (2022), https://dailymed.nlm.nih.gov.

[10] Incyte Biosciences Distribution B.V , “Opzelura 15 mg/g Cream: EU Summary of Product Characteristics,” (2023), https://www.ema.europa.eu.

[11] MHRA , “Grants Marketing Authorisation for Opzelura (Ruxolitinib) Cream for the Treatment of Non‐Segmental Vitiligo With Facial Involvement in Adults and Adolescents,” (2023), Business Wire.

[12] L. Bokhari and R. Sinclair , “Treatment of Alopecia Universalis With Topical Janus Kinase Inhibitors ‐ a Double Blind, Placebo, and Active Controlled Pilot Study,” International Journal of Dermatology 57 (2018): 1464–1470. 30160787 10.1111/ijd.14192

[13] C. B. Bayart , K. L. DeNiro , L. Brichta , B. G. Craiglow , and R. Sidbury , “Topical Janus Kinase Inhibitors for the Treatment of Pediatric Alopecia Areata,” Journal of the American Academy of Dermatology 77 (2017): 167–170. 28619556 10.1016/j.jaad.2017.03.024

[14] A. Alkattan , A. Alzaher , D. Alhabib , et al., “An Evaluation of the Recently Approved Drugs for Treating Atopic Dermatitis in the Context of Their Safety and Efficacy: A Systematic Review and Meta‐Analysis,” Expert Review of Clinical Immunology 21 (2024): 1–11. 39663577 10.1080/1744666X.2024.2435657

[15] S. J. Lax , E. Van Vogt , B. Candy , et al., “Topical Anti‐Inflammatory Treatments for Eczema: A Cochrane Systematic Review and Network Meta‐Analysis,” Clinical and Experimental Allergy 54 (2024): 960–972. 39219446 10.1111/cea.14556 PMC11629051

[16] L. A. McGuinness and J. P. T. Higgins , “Risk‐of‐Bias VISualization (Robvis): An R Package and Shiny Web App for Visualizing Risk‐of‐Bias Assessments,” Research Synthesis Methods 12 (2021): 55–61. 32336025 10.1002/jrsm.1411

[17] E. A. Olsen , D. Kornacki , K. Sun , and M. K. Hordinsky , “Ruxolitinib Cream for the Treatment of Patients With Alopecia Areata: A 2‐Part, Double‐Blind, Randomized, Vehicle‐Controlled Phase 2 Study,” Journal of the American Academy of Dermatology 82 (2020): 412–419. 31622643 10.1016/j.jaad.2019.10.016

[18] N. Punwani , P. Scherle , R. Flores , et al., “Preliminary Clinical Activity of a Topical JAK1/2 Inhibitor in the Treatment of Psoriasis,” Journal of the American Academy of Dermatology 67 (2012): 658–664. 22281165 10.1016/j.jaad.2011.12.018

[19] B. G. Craiglow , D. Tavares , and B. A. King , “Topical Ruxolitinib for the Treatment of Alopecia Universalis,” JAMA Dermatology 152 (2016): 490–491. 26649829 10.1001/jamadermatol.2015.4445

[20] M. Deeb and R. A. Beach , “A Case of Topical Ruxolitinib Treatment Failure in Alopecia Areata,” Journal of Cutaneous Medicine and Surgery 21 (2017): 562–563. 28635319 10.1177/1203475417716363

[21] Y. Tembunde and C. Kindred , “Ruxolitinib 1.5% Topical Cream for the Treatment of Pediatric Alopecia Areata,” Journal of Drugs in Dermatology 23 (2024): 378–379. 38709705 10.36849/JDD.7782

[22] T. Yan , T. Wang , M. Tang , and N. Liu , “Comparative Efficacy and Safety of JAK Inhibitors in the Treatment of Moderate‐to‐Severe Alopecia Areata: A Systematic Review and Network Meta‐Analysis,” Frontiers in Pharmacology 15 (2024): 1372810. 38659584 10.3389/fphar.2024.1372810 PMC11039836

[23] R. D. Haughton , S. M. Herbert , A. Ji‐Xu , L. Downing , S. P. Raychaudhuri , and E. Maverakis , “Janus Kinase Inhibitors for Alopecia Areata: A Narrative Review,” Indian Journal of Dermatology, Venereology and Leprology 89 (2023): 799–806. 37436019 10.25259/IJDVL_1093_2022

[24] O. Kwon , M. M. Senna , R. Sinclair , et al., “Efficacy and Safety of Baricitinib in Patients With Severe Alopecia Areata Over 52 Weeks of Continuous Therapy in Two Phase III Trials (BRAVE‐AA1 and BRAVE‐AA2),” American Journal of Clinical Dermatology 24 (2023): 443–451. 36855020 10.1007/s40257-023-00764-w PMC9974384

[25] M. Lensing and A. Jabbari , “An Overview of JAK/STAT Pathways and JAK Inhibition in Alopecia Areata,” Frontiers in Immunology 13 (2022): 955035. 36110853 10.3389/fimmu.2022.955035 PMC9470217

[26] S. B. Chikhalkar , S. Prasanna , and T. Vishwanath , “Efficacy and Safety of Topical Tofacitinib for the Treatment of Alopecia Areata,” Indian Dermatology Online Journal 15 (2024): 624–629. 39050046 10.4103/idoj.idoj_535_23 PMC11265738

[27] B. G. Craiglow , “Topical Tofacitinib Solution for the Treatment of Alopecia Areata Affecting Eyelashes,” JAAD Case Reports 4 (2018): 988–989. 30417059 10.1016/j.jdcr.2018.07.018 PMC6218694

[28] M. W. Cheng , A. Kehl , S. Worswick , and C. Goh , “Successful Treatment of Severe Alopecia Areata With Oral or Topical Tofacitinib,” Journal of Drugs in Dermatology 17 (2018): 800–803. 30005104

[29] L. Y. Liu , B. G. Craiglow , and B. A. King , “Tofacitinib 2% Ointment, a Topical Janus Kinase Inhibitor, for the Treatment of Alopecia Areata: A Pilot Study of 10 Patients,” Journal of the American Academy of Dermatology 78 (2018): 403–404.e1. 29108908 10.1016/j.jaad.2017.10.043

[30] D. Desai , A. Nohria , K. Lo Sicco , and J. Shapiro , “The Use of Topical Ruxolitinib 1.5% Cream in Frontal Fibrosing Alopecia: A Case Report,” JAAD Case Reports 50 (2024): 141–143. 39148634 10.1016/j.jdcr.2024.04.034 PMC11326510

[31] M. Nasimi and M. S. Ansari , “JAK Inhibitors in the Treatment of Lichen Planopilaris,” Skin Appendage Disorders 10 (2024): 10–17. 38313572 10.1159/000534631 PMC10836856

[32] M. L. Porriño‐Bustamante , M. A. Fernández‐Pugnaire , and S. Arias‐Santiago , “Frontal Fibrosing Alopecia: A Review,” Journal of Clinical Medicine 10 (2021): 1805. 33919069 10.3390/jcm10091805 PMC8122646

[33] C. C. Yang , T. Khanna , B. Sallee , A. M. Christiano , and L. A. Bordone , “Tofacitinib for the Treatment of Lichen Planopilaris: A Case Series,” Dermatologic Therapy 31 (2018): e12656. 30264512 10.1111/dth.12656 PMC6585740

[34] A. C. Katoulis , K. Diamanti , D. Sgouros , et al., “Frontal Fibrosing Alopecia and Vitiligo: Coexistence or True Association?,” Skin Appendage Disorders 2 (2017): 152–155. 28232924 10.1159/000452449 PMC5264360

[35] A. C. Katoulis , K. Diamanti , D. Sgouros , et al., “Frontal Fibrosing Alopecia: Is the Melanocyte of the Upper Hair Follicle the Antigenic Target?,” International Journal of Dermatology 57 (2018): e37–e38. 29732556 10.1111/ijd.14021

[36] C. M. Brumfiel , M. H. Patel , K. J. Severson , et al., “Ruxolitinib Cream in the Treatment of Cutaneous Lichen Planus: A Prospective, Open‐Label Study,” Journal of Investigative Dermatology 142 (2022): 2109–2116.e4. 35131254 10.1016/j.jid.2022.01.015

[37] H. L. Cornman , E. Wei , J. Manjunath , et al., “Recalcitrant Lichen Planus Pigmentosus Treated With Topical Ruxolitinib,” JAAD Case Reports 42 (2023): 84–86. 38156096 10.1016/j.jdcr.2023.10.012 PMC10753049

[38] M. Min , A. S. Dulai , and R. K. Sivamani , “Recalcitrant Multi‐Variant Lichen Planus Successfully Treated With Oral Baricitinib and Topical Ruxolitinib Cream,” Dermatology Online Journal 30 (2024). 10.5070/D330363864 39090037

[39] S. Shao , L. C. Tsoi , M. K. Sarkar , et al., “IFN‐γ Enhances Cell‐Mediated Cytotoxicity Against Keratinocytes via JAK2/STAT1 in Lichen Planus,” Science Translational Medicine 11 (2019): eaav7561. 31554739 10.1126/scitranslmed.aav7561 PMC7285657

[40] A. Motamed‐Sanaye , Y. F. Khazaee , M. Shokrgozar , M. Alishahi , N. Ahramiyanpour , and M. Amani , “JAK Inhibitors in Lichen Planus: A Review of Pathogenesis and Treatments,” Journal of Dermatological Treatment 33 (2022): 3098–3103. 35997540 10.1080/09546634.2022.2116926

[41] M. P. Zundell , R. Al‐Dehneem , T. Song , J. Yousif , and A. B. Gottlieb , “Novel Clinical Applications of Topical Ruxolitinib: A Case Series,” Journal of Drugs in Dermatology 23 (2024): 188–190. 38443119 10.36849/jdd.7696

[42] M. Corazza , N. Schettini , P. Zedde , and A. Borghi , “Vulvar Lichen Sclerosus From Pathophysiology to Therapeutic Approaches: Evidence and Prospects,” Biomedicine 9 (2021): 950. 10.3390/biomedicines9080950 PMC8394941 34440154

[43] S. R. Kim , A. Charos , W. Damsky , P. Heald , M. Girardi , and B. A. King , “Treatment of Generalized Deep Morphea and Eosinophilic Fasciitis With the Janus Kinase Inhibitor Tofacitinib,” JAAD Case Reports 4 (2018): 443–445. 29984277 10.1016/j.jdcr.2017.12.003 PMC6031588

[44] S. McGaugh , P. Kallis , A. De Benedetto , and R. M. Thomas , “Janus Kinase Inhibitors for Treatment of Morphea and Systemic Sclerosis: A Literature Review,” Dermatologic Therapy 35 (2022): e15437. 35278019 10.1111/dth.15437

[45] W. Wang , S. Bhattacharyya , R. G. Marangoni , et al., “The JAK/STAT Pathway Is Activated in Systemic Sclerosis and Is Effectively Targeted by Tofacitinib,” Journal of Scleroderma and Related Disorders 5 (2020): 40–50. 35382402 10.1177/2397198319865367 PMC8922593

[46] A. J. Piontkowski , N. Wei , A. Mumtaz , and N. Gulati , “Ruxolitinib Cream for the Treatment of Granuloma Annulare,” JAAD Case Reports 50 (2024): 62–64. 39050923 10.1016/j.jdcr.2024.05.030 PMC11266857

[47] W. Damsky , D. Thakral , M. K. McGeary , J. Leventhal , A. Galan , and B. King , “Janus Kinase Inhibition Induces Disease Remission in Cutaneous Sarcoidosis and Granuloma Annulare,” Journal of the American Academy of Dermatology 82 (2020): 612–621. 31185230 10.1016/j.jaad.2019.05.098 PMC7590533

[48] M. S. Min , J. Wu , H. He , et al., “Granuloma Annulare Skin Profile Shows Activation of T‐Helper Cell Type 1, T‐Helper Cell Type 2, and Janus Kinase Pathways,” Journal of the American Academy of Dermatology 83 (2020): 63–70. 31870914 10.1016/j.jaad.2019.12.028

[49] N. C. Gorham , J. Jacobs , and S. Z. Wu , “Response of Severe Lupus Miliaris Disseminatus Faciei to Treatment With Ruxolitinib Cream,” JAMA Dermatology 159 (2023): 790–791. 37099285 10.1001/jamadermatol.2023.0528

[50] D. M. Schwartz , Y. Kanno , A. Villarino , M. Ward , M. Gadina , and J. J. O'Shea , “JAK Inhibition as a Therapeutic Strategy for Immune and Inflammatory Diseases,” Nature Reviews. Drug Discovery 17 (2017): 78. 10.1038/nrd.2017.267 PMC6168198 29282366

[51] A. S. Hwang , J. A. Kechter , X. Li , et al., “Topical Ruxolitinib in the Treatment of Necrobiosis Lipoidica: A Prospective, Open‐Label Study,” Journal of Investigative Dermatology 144 (2024): 1994–2001.e4. 38417541 10.1016/j.jid.2023.11.027

[52] S. Nugent , A. J. Coromilas , J. C. English , and M. Rosenbach , “Improvement of Necrobiosis Lipoidica With Topical Ruxolitinib Cream After Prior Nonresponse to Compounded Topical Tofacitinib Cream,” JAAD Case Reports 29 (2022): 25–26. 36186415 10.1016/j.jdcr.2022.08.028 PMC9522866

[53] J. J. Lee and J. C. English , “Improvement in Ulcerative Necrobiosis Lipoidica After Janus Kinase‐Inhibitor Therapy for Polycythemia Vera,” JAMA Dermatology 154 (2018): 733–734. 29800055 10.1001/jamadermatol.2018.0756

[54] W. Damsky , K. Singh , A. Galan , and B. King , “Treatment of Necrobiosis Lipoidica With Combination Janus Kinase Inhibition and Intralesional Corticosteroid,” JAAD Case Reports 6 (2020): 133–135. 32021895 10.1016/j.jdcr.2019.11.016 PMC6994271

[55] M.‐A. Barbet‐Massin , V. Rigalleau , P. Blanco , et al., “Remission of Necrobiosis Lipoidica Diabeticorum With a JAK1/2 Inhibitor: A Case Report,” Diabetes & Metabolism 47, no. 4 (2021): 101143, 10.1016/j.diabet.2020.01.001. 31981714

[56] S. Janßen and T. M. Jansen , “Ulcerated Necrobiosis Lipoidica Successfully Treated With Tofacitinib,” International Journal of Dermatology 61 (2022): 739–741. 34783006 10.1111/ijd.15960

[57] J. S. Smith , M. J. Woodbury , and J. F. Merola , “Ruxolitinib Cream for the Treatment of Cutaneous Sarcoidosis,” JAAD Case Reports 38 (2023): 111–112. 37521191 10.1016/j.jdcr.2023.05.032 PMC10372038

[58] R. Talty , W. Damsky , and B. King , “Treatment of Cutaneous Sarcoidosis With Tofacitinib: A Case Report and Review of Evidence for Janus Kinase Inhibition in Sarcoidosis,” JAAD Case Reports 16 (2021): 62–64. 34522749 10.1016/j.jdcr.2021.08.012 PMC8427262

[59] M. Alam , V. Fang , and M. Rosenbach , “Treatment of Cutaneous Sarcoidosis With Tofacitinib 2% Ointment and Extra Virgin Olive Oil,” JAAD Case Reports 9 (2021): 1–3. 33598514 10.1016/j.jdcr.2020.12.021 PMC7868739

[60] A. Wong , S. Stahly , J. Kieffer , C. Dunn , and R. Nathoo , “Topical Ruxolitinib for the Treatment of Granuloma Faciale,” JAAD Case Reports 36 (2023): 73–74. 37250006 10.1016/j.jdcr.2023.04.013 PMC10220211

[61] T. Nakahara , Y. Moroi , A. Tashiro , H. Kiryu , and M. Furue , “The Interaction of Inflammatory Cells in Granuloma Faciale,” Dermatology Reports 2 (2010): e17. 25386252 10.4081/dr.2010.e17 PMC4211470

[62] B. R. Smoller and J. Bortz , “Immunophenotypic Analysis Suggests That Granuloma Faciale Is a γ‐Interferon‐Mediated Process,” Journal of Cutaneous Pathology 20 (1993): 442–446. 7507947 10.1111/j.1600-0560.1993.tb00668.x

[63] M. Ferrarini , D. J. Helfrich , E. R. Walker , T. A. Medsger , and T. L. Whiteside , “Scleromyxedema Serum Increases Proliferation but Not the Glycosaminoglycan Synthesis of Dermal Fibroblasts,” Journal of Rheumatology 16 (1989): 837–841. 2778769

[64] D. Zhang , K. Sable , A. Miller , M. Hinshaw , T. Schmidt , and B. E. Shields , “Granulomatous Variant of Scleromyxedema Successfully Treated With Topical Ruxolitinib, Dapsone and Intravenous Immunoglobulin,” JAAD Case Reports 42 (2023): 78–83. 38156097 10.1016/j.jdcr.2023.10.010 PMC10753040

[65] A. R. Furtunescu , S. R. Georgescu , M. Tampa , and C. Matei , “Inhibition of the JAK‐STAT Pathway in the Treatment of Psoriasis: A Review of the Literature,” International Journal of Molecular Sciences 25 (2024): 4681. 38731900 10.3390/ijms25094681 PMC11083046

[66] M. Pj , “The JAK‐STAT Signaling Pathway: Input and Output Integration,” Journal of Immunology 178, no. 5 (2007): 2623–2629, 10.4049/jimmunol.178.5.2623. 17312100

[67] Y. Han , Y. R. Woo , S. H. Cho , J. D. Lee , and H. S. Kim , “Itch and Janus Kinase Inhibitors,” Acta Dermato‐Venereologica 103 (2023): adv00869. 36789757 10.2340/actadv.v103.5346 PMC9944301

[68] J. Shin and M. Leger , “Tattoo Pruritus Successfully Treated With Ruxolitinib: A Case Series,” JAAD Case Reports 47 (2024): 107–109. 38699580 10.1016/j.jdcr.2024.02.020 PMC11063527

[69] J. J. Park , A. J. Little , and M. D. Vesely , “Treatment of Cutaneous Lupus With Topical Ruxolitinib Cream,” JAAD Case Reports 28 (2022): 133–135. 36159722 10.1016/j.jdcr.2022.08.038 PMC9494033

[70] T. Fetter , P. Smith , T. Guel , C. Braegelmann , T. Bieber , and J. Wenzel , “Selective Janus Kinase 1 Inhibition Is a Promising Therapeutic Approach for Lupus Erythematosus Skin Lesions,” Frontiers in Immunology 11 (2020): 344. 32194562 10.3389/fimmu.2020.00344 PMC7064060

[71] D. R. Mazori , M. S. Min , B. Kassamali , et al., “Use of Tofacitinib, 2%, Ointment for Periorbital Discoid Lupus Erythematosus,” JAMA Dermatology 157 (2021): 880–882. 34076672 10.1001/jamadermatol.2021.1198

[72] E. Pope , E. Kowalski , and F. Tausk , “Topical Ruxolitinib in the Treatment of Refractory Facial Seborrheic Dermatitis,” JAAD Case Reports 24 (2022): 59–60. 35619595 10.1016/j.jdcr.2022.04.003 PMC9127103

[73] M. Teklu and H. J. Chung , “Letter in Response to the Case Report ‘Topical Ruxolitinib in the Treatment of Refractory Facial Seborrheic Dermatitis’,” JAAD Case Reports 42 (2023): 45–46. 38034367 10.1016/j.jdcr.2023.09.038 PMC10684363

[74] J. A. Adalsteinsson , S. Kaushik , S. Muzumdar , E. Guttman‐Yassky , and J. Ungar , “An Update on the Microbiology, Immunology and Genetics of Seborrheic Dermatitis,” Experimental Dermatology 29 (2020): 481–489. 32125725 10.1111/exd.14091

[75] S. S. Tran , B. Ungar , and P. M. Brunner , “Treatment of Granulomatous Perioral Dermatitis With 1.5% Topical Ruxolitinib Cream,” JAAD Case Reports 47 (2024): 1–3. 38576899 10.1016/j.jdcr.2024.02.022 PMC10990702

[76] D. H. Zarowin , L. Provini , and R. J. Antaya , “Topical Ruxolitinib for Facial Blaschkitis in an Adolescent,” Pediatric Dermatology 41, no. 5 (2024): 938–939, 10.1111/pde.15640. 38716713

[77] C. S. L. Müller , R. Schmaltz , T. Vogt , and C. Pföhler , “Lichen Striatus and Blaschkitis: Reappraisal of the Concept of Blaschkolinear Dermatoses,” British Journal of Dermatology 164 (2011): 257–262. 20849467 10.1111/j.1365-2133.2010.10053.x

[78] C. M. Powers , H. Verma , J. Orloff , et al., “Use of a Topical Janus Kinase Inhibitor in Immune Checkpoint Inhibitor‐Induced Eczematous Reaction: A Case Report,” Journal of Dermatological Treatment 35 (2024): 2336118. 38565207 10.1080/09546634.2024.2336118

[79] R. Reschke , J. W. Shapiro , J. Yu , et al., “Checkpoint Blockade‐Induced Dermatitis and Colitis Are Dominated by Tissue‐Resident Memory T Cells and Th1/Tc1 Cytokines,” Cancer Immunology Research 10 (2022): 1167–1174. 35977003 10.1158/2326-6066.CIR-22-0362 PMC9530647

[80] J. Khang , J. M. Yardman‐Frank , L.‐C. Chen , and H. J. Chung , “Recalcitrant Hailey‐Hailey Disease Successfully Treated With Topical Ruxolitinib Cream and Dupilumab,” JAAD Case Reports 42 (2023): 56–58. 38058412 10.1016/j.jdcr.2023.10.004 PMC10696304

[81] R. Sudbrak , J. Brown , C. Dobson‐Stone , et al., “Hailey‐Hailey Disease Is Caused by Mutations in ATP2C1 Encoding a Novel Ca^2+^ Pump,” Human Molecular Genetics 9 (2000): 1131–1140. 10767338 10.1093/hmg/9.7.1131

[82] Y. Li , Y. Jiang , and J. Sun , “Improvement of Hailey‐Hailey Disease With Abrocitinib,” Clinical and Experimental Dermatology 48 (2023): 532–533. 36723952 10.1093/ced/llad023

[83] M. Shea , E. Grinich , and E. Simpson , “Topical Steroid Withdrawal Treated With Ruxolitinib Cream,” JAAD Case Reports 48 (2024): 5–7. 38745830 10.1016/j.jdcr.2024.03.011 PMC11091456

[84] S. K. Stacey and M. McEleney , “Topical Corticosteroids: Choice and Application,” American Family Physician 103 (2021): 337–343. 33719380

[85] F. Spano and J. C. Donovan , “Alopecia Areata: Part 1: Pathogenesis, Diagnosis, and Prognosis,” Canadian Family Physician 61 (2015): 751–755. 26371097 PMC4569104

[86] B. Tekin , F. Xie , and J. S. Lehman , “Lichen Planus: What Is New in Diagnosis and Treatment?,” American Journal of Clinical Dermatology 25 (2024): 735–764. 38982032 10.1007/s40257-024-00878-9

[87] A. Abduelmula , A. Bagit , A. Mufti , K. C. Y. Yeung , and J. Yeung , “The Use of Janus Kinase Inhibitors for Lichen Planus: An Evidence‐Based Review,” Journal of Cutaneous Medicine and Surgery 27 (2023): 271–276. 36815857 10.1177/12034754231156100 PMC10291104

[88] V. Di Lernia , “Targeting the IFN‐γ/CXCL10 Pathway in Lichen Planus,” Medical Hypotheses 92 (2016): 60–61. 27241258 10.1016/j.mehy.2016.04.042

[89] S. Chapman , M. Kwa , L. S. Gold , and H. W. Lim , “Janus Kinase Inhibitors in Dermatology: Part I. A Comprehensive Review,” Journal of the American Academy of Dermatology 86 (2022): 406–413. 34246698 10.1016/j.jaad.2021.07.002

[90] S. Chapman , L. S. Gold , and H. W. Lim , “Janus Kinase Inhibitors in Dermatology: Part II. A Comprehensive Review,” Journal of the American Academy of Dermatology 86 (2022): 414–422. 34246698 10.1016/j.jaad.2021.07.002

